# Supplementary material for: Conserved collateral antibiotic susceptibility networks in diverse clinical strains of Escherichia coli
Source: Nat Commun. 2018 Sep 10;9:3673. doi: 10.1038/s41467-018-06143-y (PMC6131505; doi:10.1038/s41467-018-06143-y)
Supplement: Supplementary file 1 — Supplementary Information [file 41467_2018_6143_MOESM1_ESM.pdf]

Supplementary Information

Conserved collateral susceptibility networks in diverse clinical strains of *Escherichia coli.*

Podnecky et al.

a

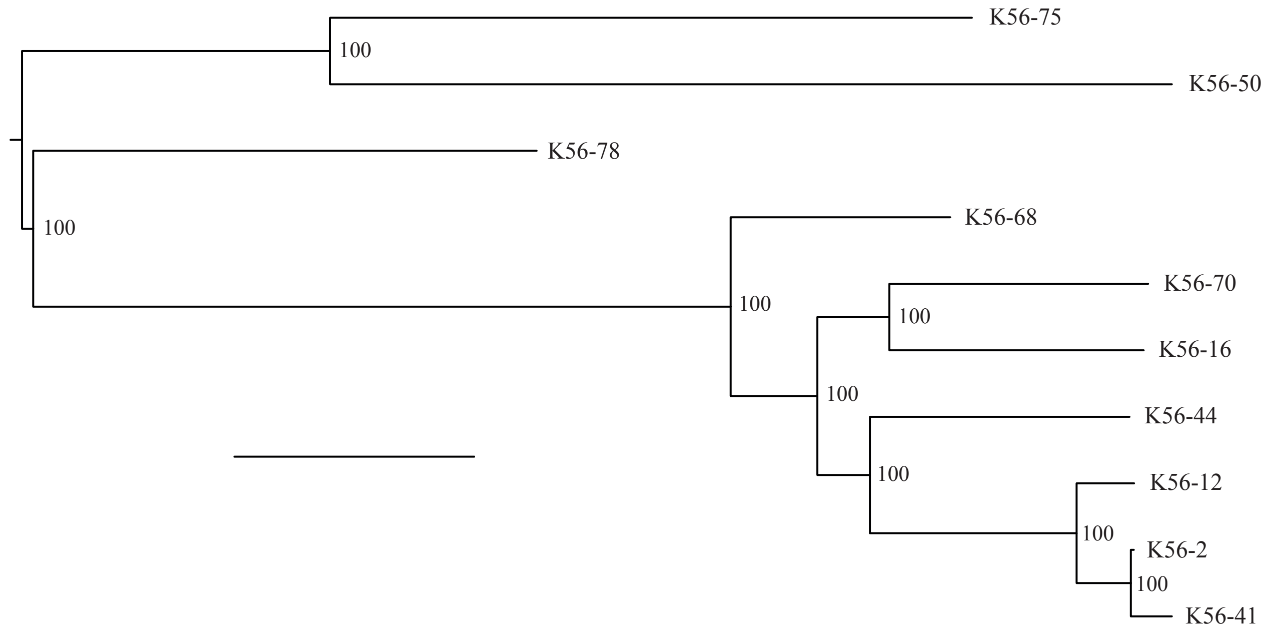

b

WT Strain	K56-2	K56-44	K56-70	K56-16	K56-78	K56-75	K56-50	K56-68	K56-12	K56-41
K56-2	0	0.008733	0.010795	0.010721	0.026755	0.034387	0.037722	0.010389	0.001913	0.000734
K56-44	0.008733	0	0.010722	0.010648	0.026682	0.034314	0.037649	0.010316	0.008736	0.009367
K56-70	0.010795	0.010722	0	0.008556	0.026992	0.034624	0.037959	0.010626	0.010798	0.011429
K56-16	0.010721	0.010648	0.008556	0	0.026918	0.03455	0.037885	0.010552	0.010724	0.011355
K56-78	0.026755	0.026682	0.026992	0.026918	0	0.024426	0.027761	0.023692	0.026758	0.027389
K56-75	0.034387	0.034314	0.034624	0.03455	0.024426	0	0.024743	0.031324	0.03439	0.035021
K56-50	0.037722	0.037649	0.037959	0.037885	0.027761	0.024743	0	0.034659	0.037725	0.038356
K56-68	0.010389	0.010316	0.010626	0.010552	0.023692	0.031324	0.034659	0	0.010392	0.011023
K56-12	0.001913	0.008736	0.010798	0.010724	0.026758	0.03439	0.037725	0.010392	0	0.002547
K56-41	0.000734	0.009367	0.011429	0.011355	0.027389	0.035021	0.038356	0.011023	0.002547	0

c

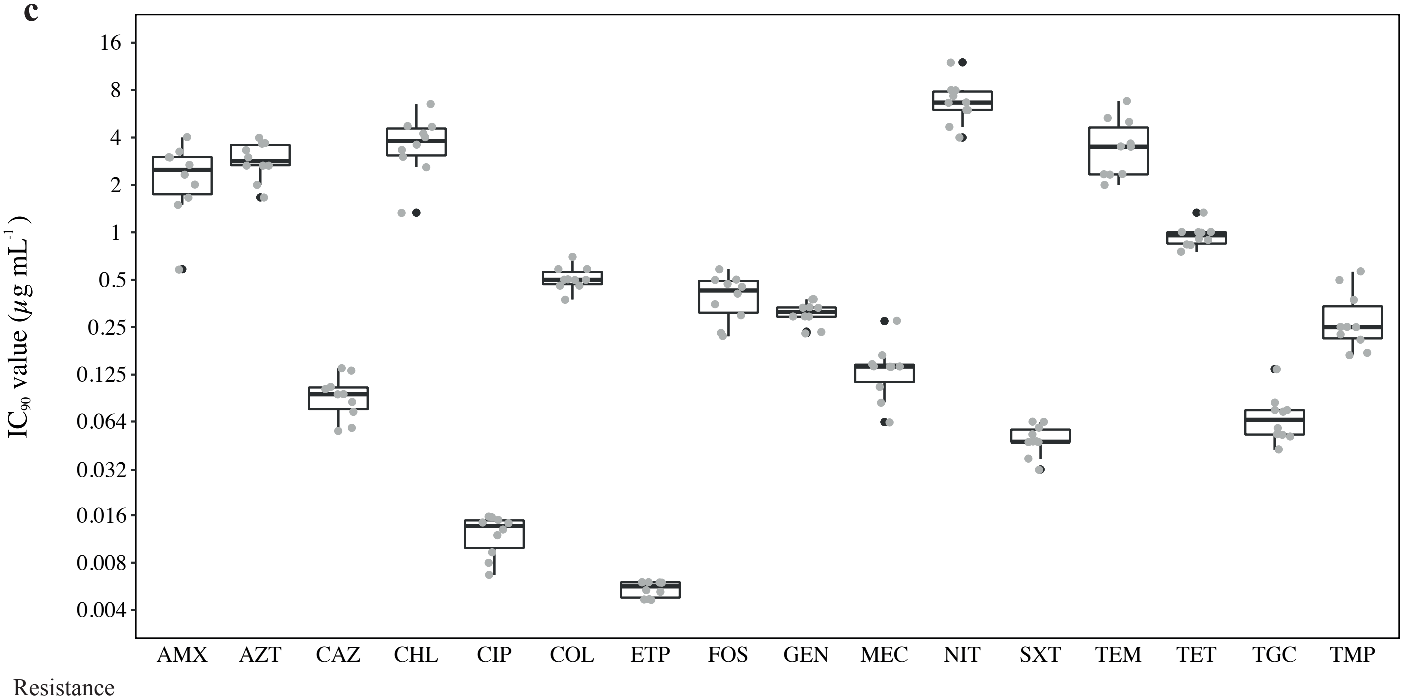

Resistance Breakpoint

AMX	AZT	CAZ	CHL	CIP	COL	ETP	FOS	GEN	MEC	NIT	SXT	TEM	TET	TGC	TMP
> 8	n/a	> 4	> 8	> 0.5	> 2	> 1	> 32	> 4	> 8	> 64	> 4	n/a	n/a	> 2	> 4

Supplementary Figure 1

Supplementary Figure 1. Characteristics of wild-type *Escherichia coli* clinical isolates. (**a**) Phylogenetic tree of the ten wild-type *E. coli* strains used in this study, based on analysis of the core protein-coding genomes (approximately 3.22 Mbp). This analysis included 100 bootstrap values, which demonstrated good support. Scale bar represents 0.004 substitutions per site. (**b**) Genetic distance between strains based on the core-genome phylogenetic analysis was calculated. Both analyses show that this is a genetically diverse collection of isolates (**a**-**b**). (**c**) The average of at least three biological replicate IC_{90} values for each wild-type *E. coli* isolate (grey dots) are shown. Summary box plots (black) of the 10 strains are overlaid showing similar susceptibilities across the strains for each of 16 antimicrobials tested. None of the wild-type strains had IC_{90} values above clinical breakpoints. EUCAST clinical resistance breakpoints in $\mu g\ mL^{-1}$ for Enterobacteriaceae[1] are shown below the *x*-axis, but were not available (n/a) for azithromycin, temocillin and tetracycline.

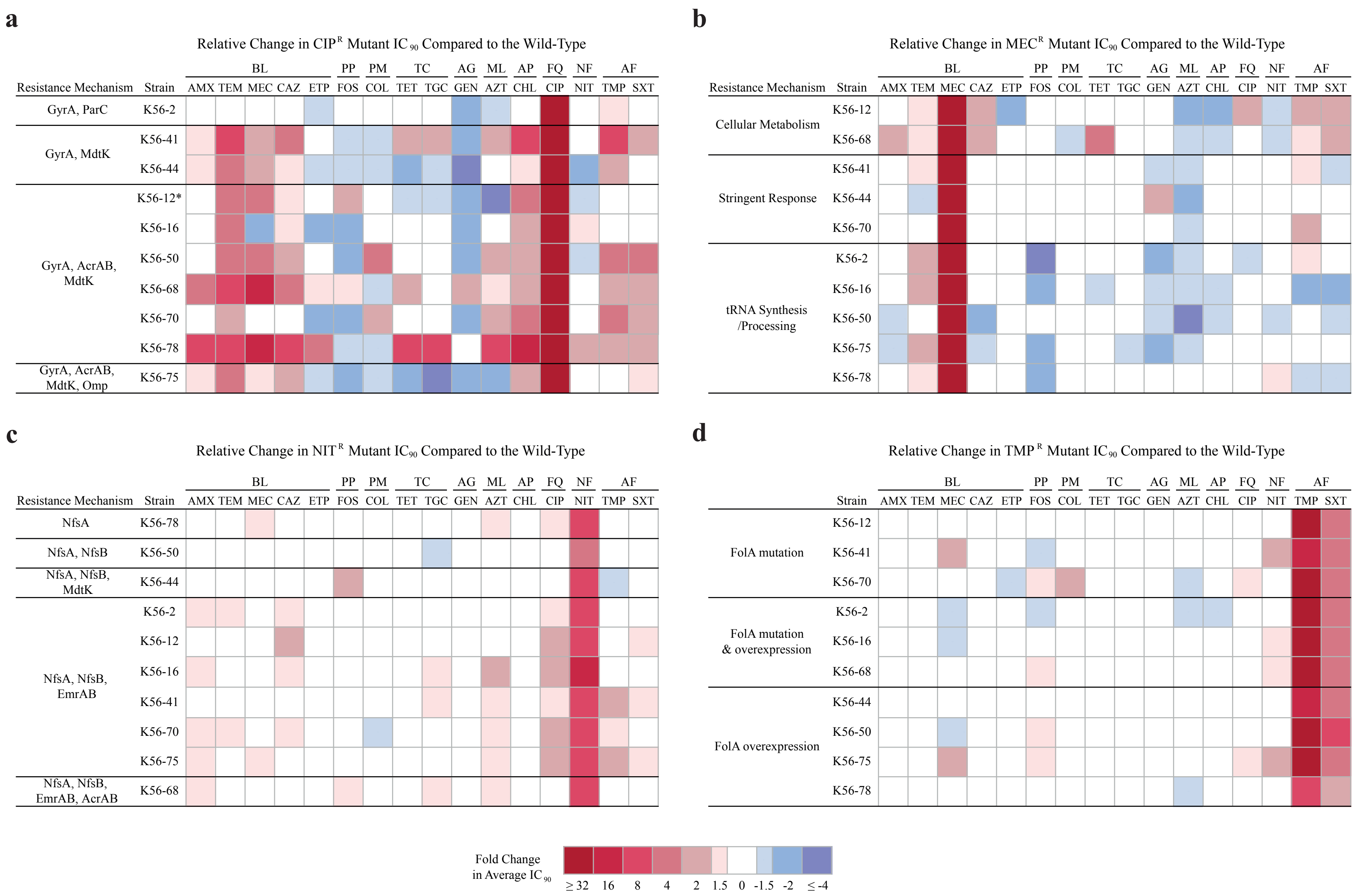

Supplementary Figure 2

Supplementary Figure 2. Collateral changes in susceptibility of 40 antimicrobial resistant mutants to 16 antimicrobials. Inhibitory concentration ≥ 90% (IC_{90}) measurements for antimicrobial resistant mutants and their respective wild-type strains were tested in at least three biological replicates and averaged. The relative fold change of the average IC_{90} of the resistant mutants compared to the wild-type is displayed in the above heat maps, where an increase in IC_{90} (red shades) indicates cross-resistance or resistance to the drug used for mutant selection, and a decrease in IC_{90} (blue shades) indicates collateral sensitivity. Increasing color intensity indicates greater collateral responses, while white indicates no change in susceptibility. Resistant mutants were ordered according to their putative resistance mechanism as determined by whole-genome sequencing (Supplementary Data 1-4). Collateral responses were most frequent among ciprofloxacin-resistant mutants, where IC_{90} values were above clinical breakpoints for six mutants to chloramphenicol and in K56-68 CIP^R to amoxicillin (**a**). Mutants resistant to mecillinam (**b**) had more collateral responses than those resistant to nitrofurantoin (**c**) and trimethoprim (**d**), however these changes were less conserved across the resistance group than was observed in the ciprofloxacin-resistant group. Abbreviations: Drug classes (BL – β-lactam, PP - phosphonic, PM – polymyxin, TC – tetracycline, AG – aminoglycoside, ML – macrolide, AP – amphenicol, FQ – fluoroquinolone, NF – nitrofuran, AF – anti-folate).

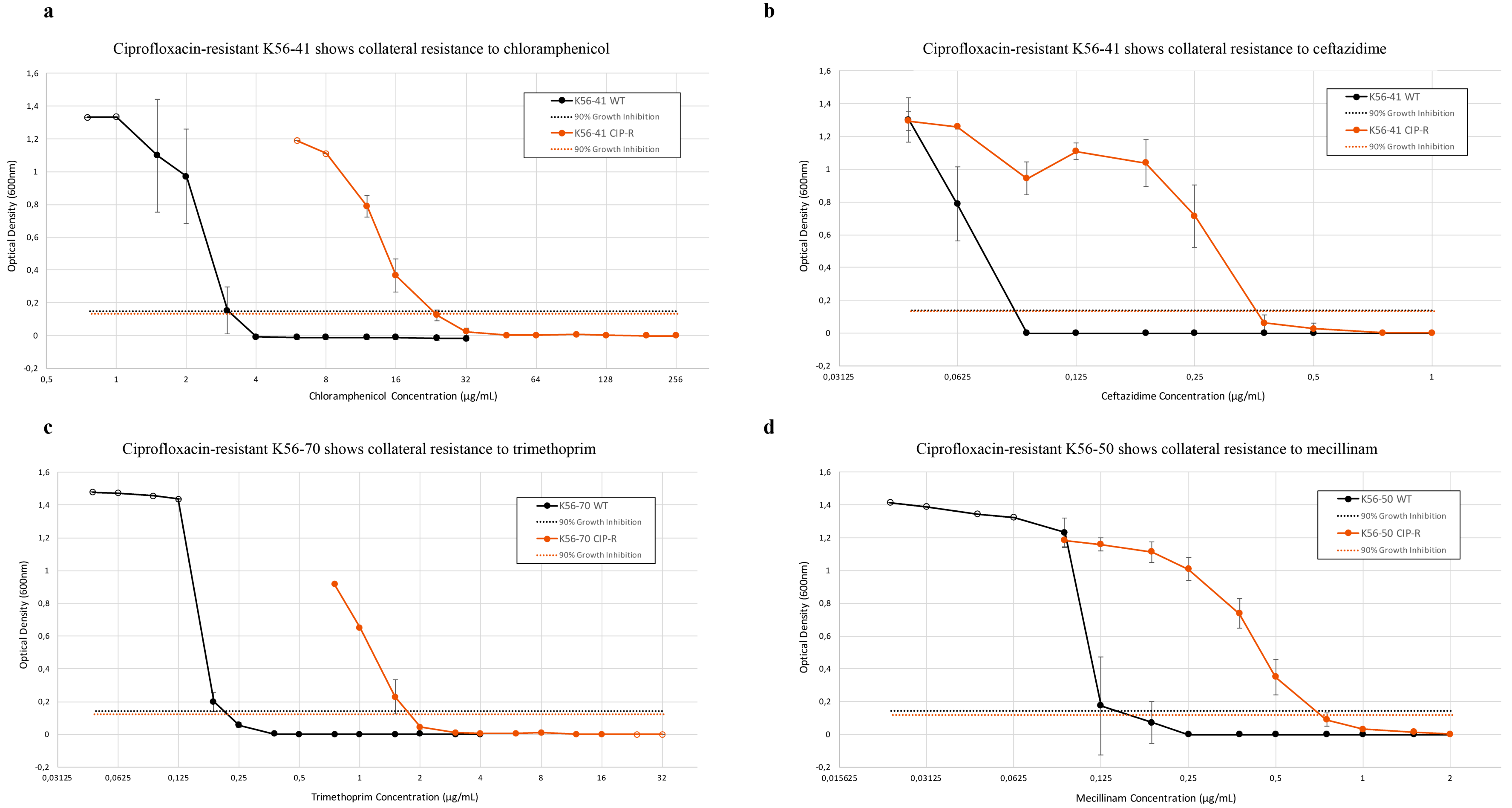

Supplementary Figure 3

e

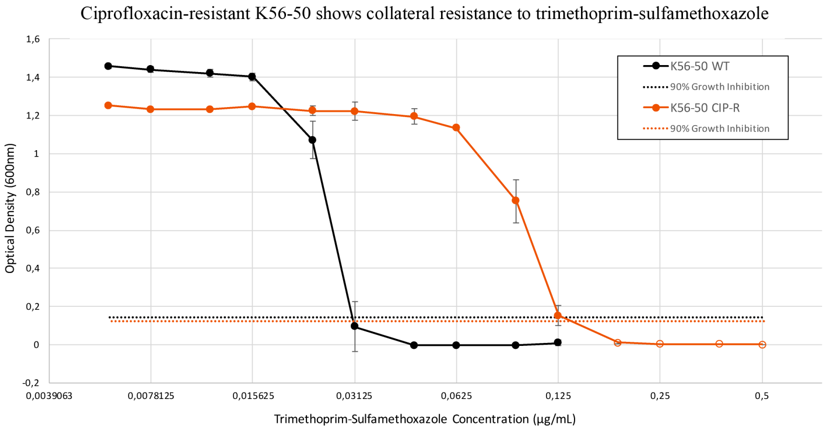

f

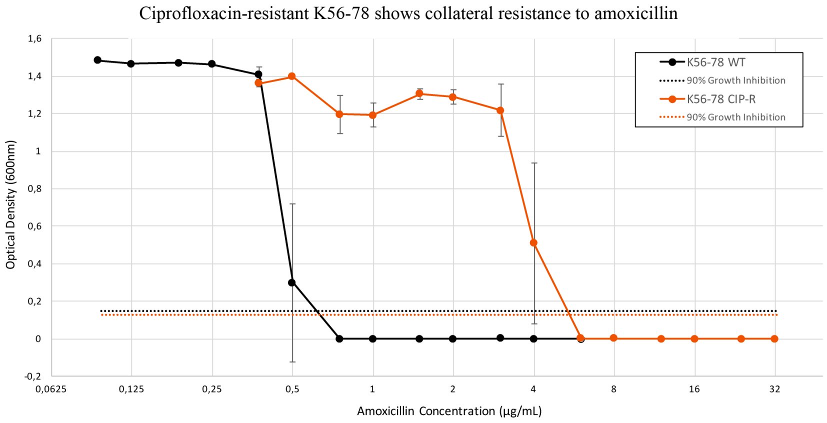

g

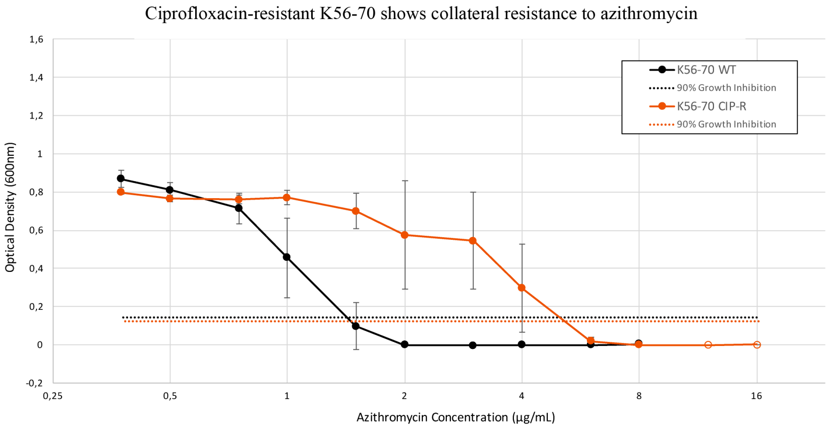

h

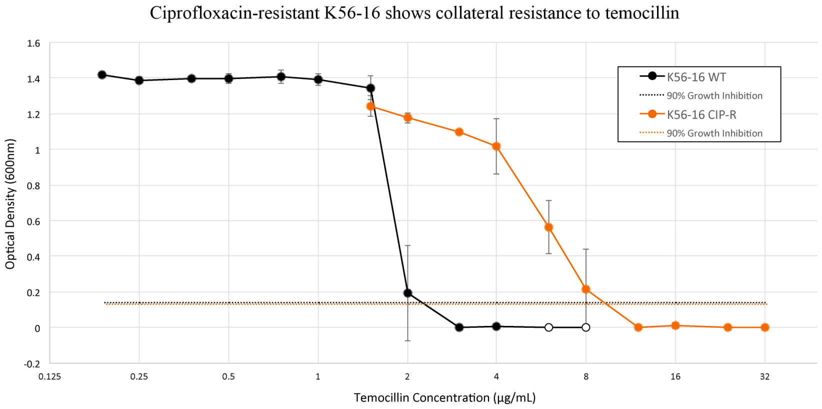

Supplementary Figure 3

i

Ciprofloxacin-resistant K56-75 shows collateral resistance to colistin

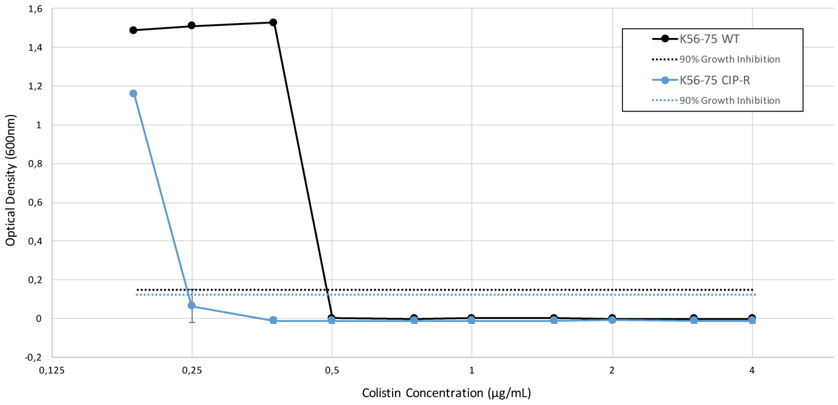

j

Ciprofloxacin-resistant K56-70 shows collateral resistance to ertapenem

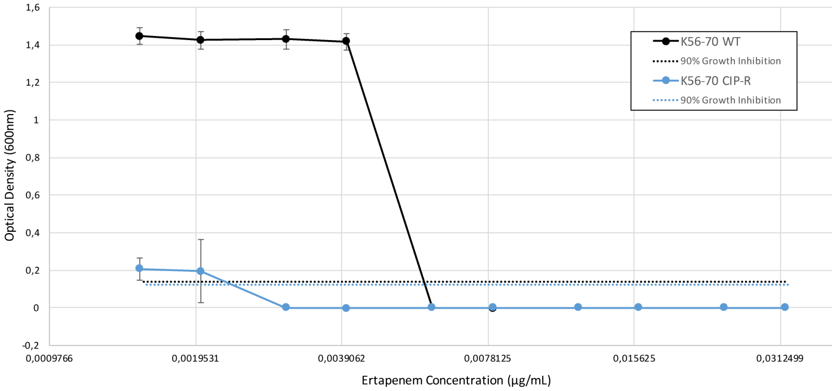

k

Ciprofloxacin-resistant K56-16 shows collateral resistance to fosfomycin

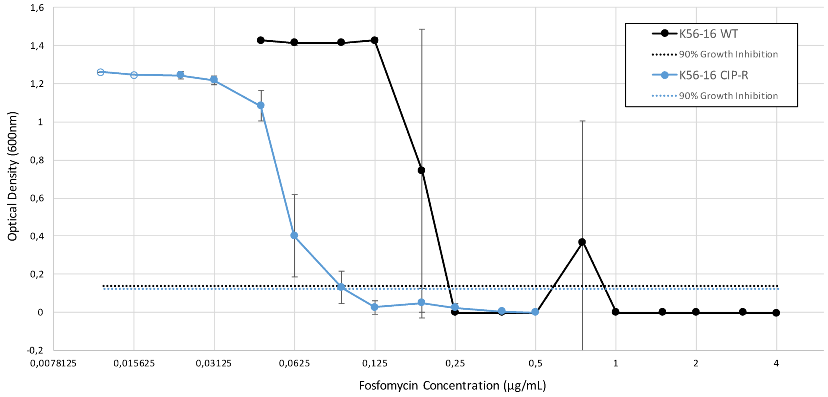

l

Ciprofloxacin-resistant K56-75 shows collateral resistance to gentamicin

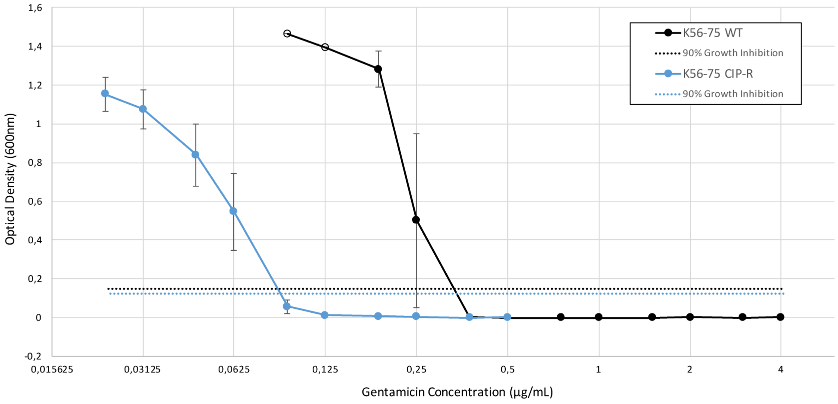

Supplementary Figure 3

m

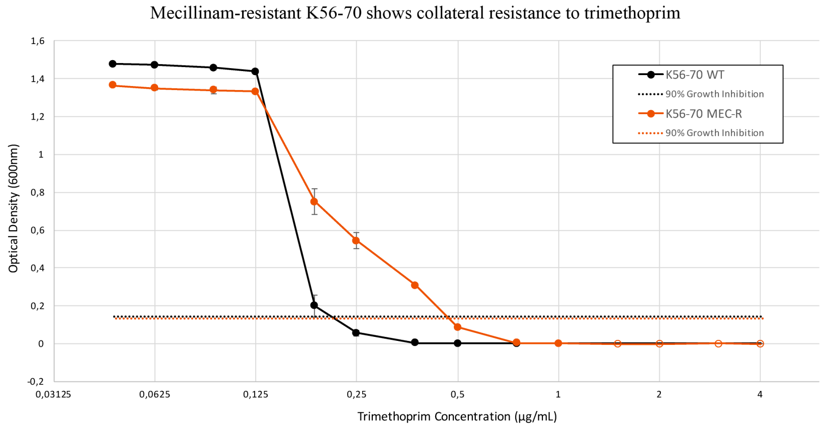

n

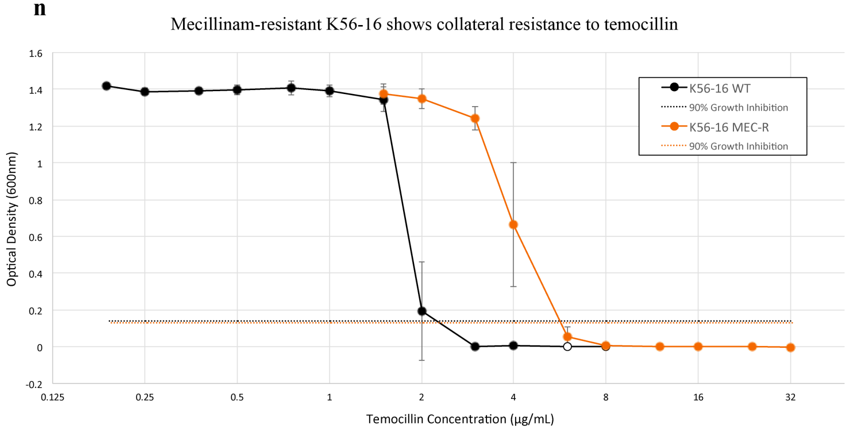

o

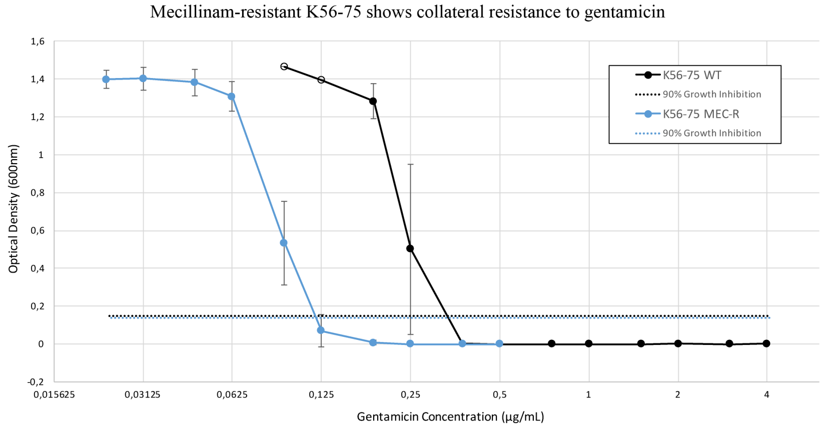

p

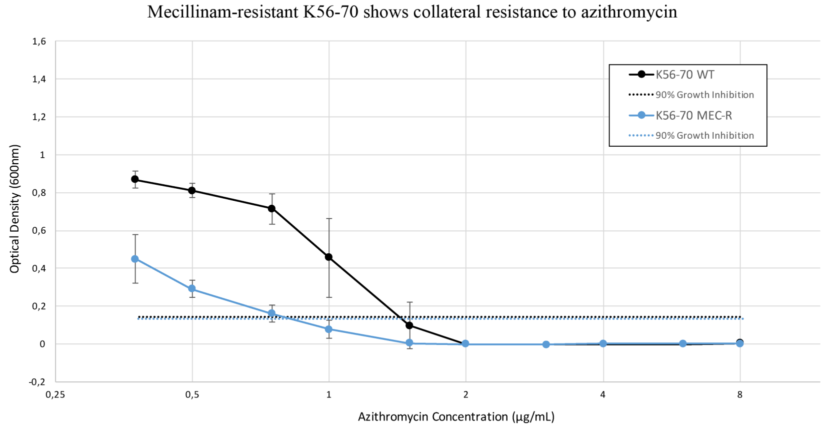

Supplementary Figure 3

q

Nitrofurantoin-resistant K56-16 shows collateral resistance to ciprofloxacin

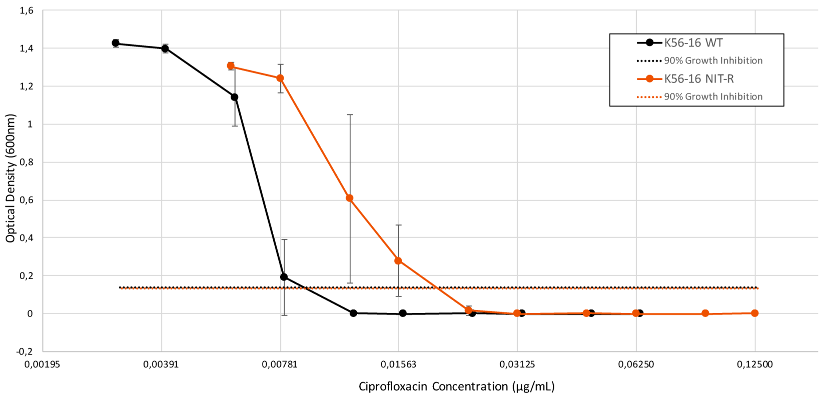

r

Nitrofurantoin-resistant K56-16 shows collateral resistance to azithromycin

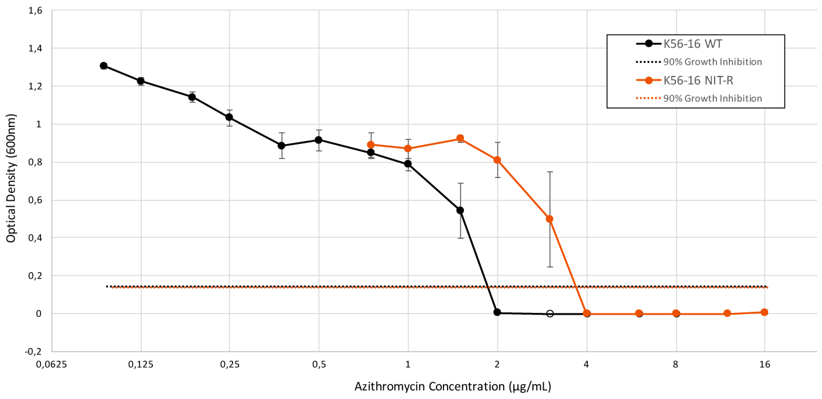

s

Nitrofurantoin-resistant K56-70 shows collateral resistance to amoxicillin

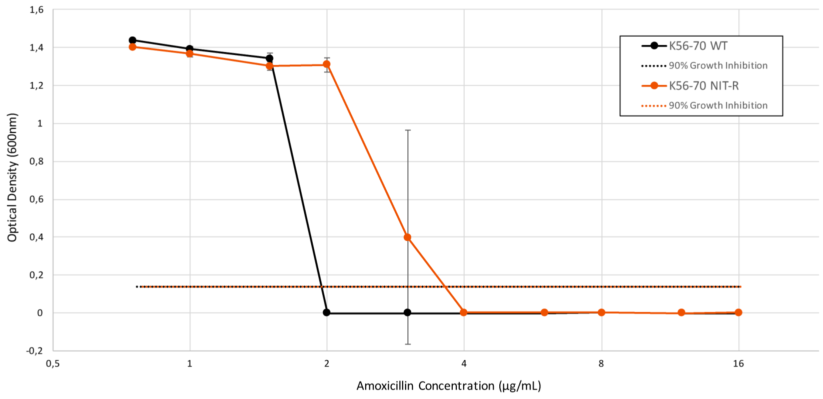

Supplementary Figure 3

Supplementary Figure 3. Dose response curves of representative strain:drug combinations. The optical density at 600 nm (OD_{600}) was measured following 18 hour shaking incubation of antimicrobial resistant mutants (colored) and respective wild-type isolates (WT; black) in Mueller-Hinton broth with varying concentrations of antimicrobial. Representative strain:drug combinations were chosen for each conserved collateral response (Fig. 1a). Closed points represent the average of at least two biological replicates and error bars indicate standard deviation (n=2-4), while open points indicate a single measurement. Dotted lines represent the OD_{600} equivalent to 90% growth inhibition (average of at least three biological replicates) of the respective strains with no antimicrobial present. The x-value at the intercept of the respective dotted and solid lines indicates the approximate average 90% inhibition concentration (IC_{90}). (**a-h**) Eight CR (red) and (**i-l**) four CS (blue) responses were observed in mutants resistant to ciprofloxacin. Analysis with fosfomycin frequently resulted in spontaneous resistance above the reproducible IC_{90} value, as shown with the wild-type K56-16 strain. Mutants resistant to mecillinam had fewer CS_{50} and CR_{50} instances; four were tested, these included (**m-n**) CR to trimethoprim and temocillin and (**o-p**) CS to gentamicin and azithromycin. Nitrofurantoin-resistant mutants (**q-s**) only showed conserved CR responses to ciprofloxacin, azithromycin and amoxicillin.

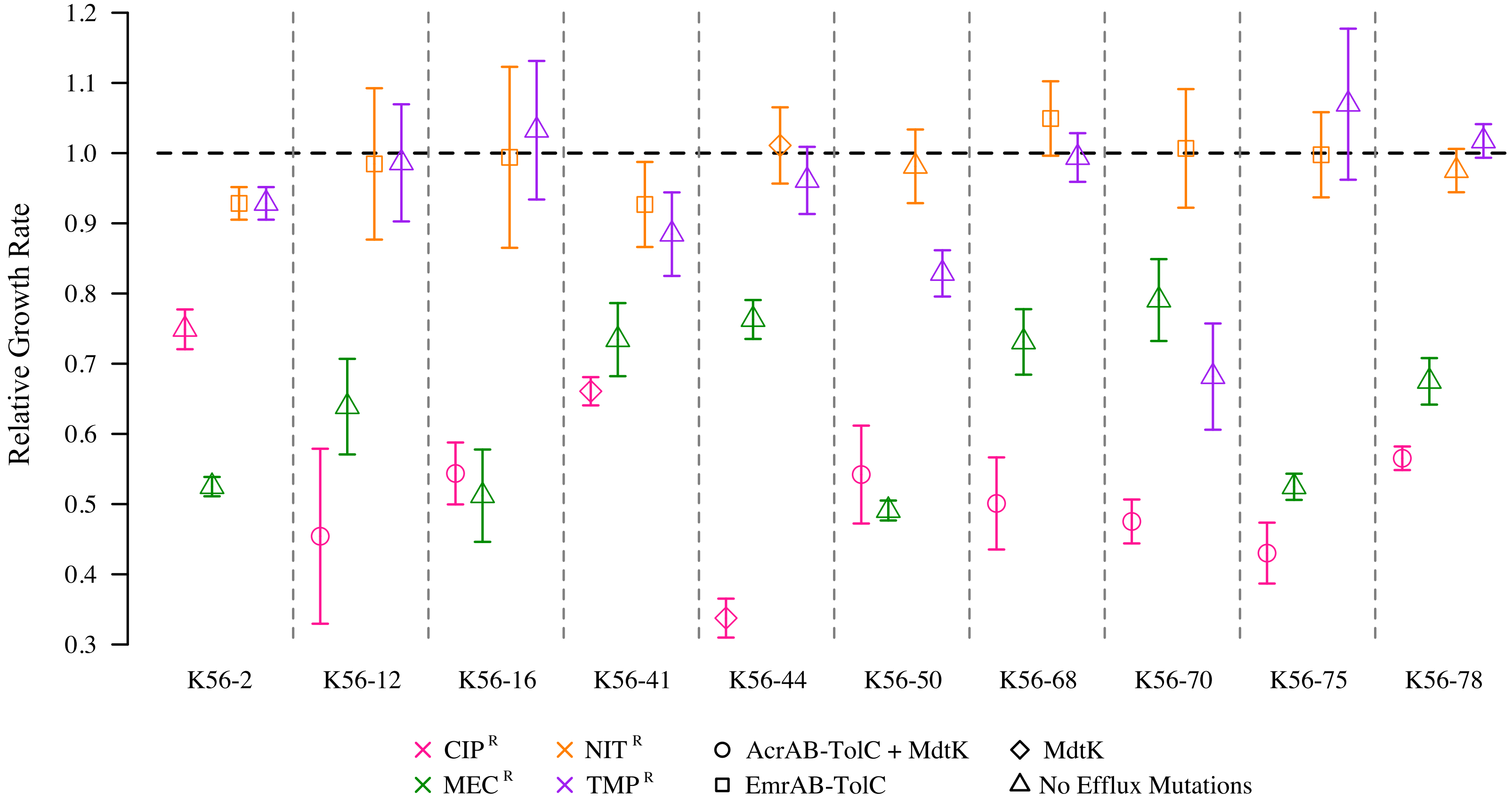

Supplementary Figure 4. Relative growth rate of antimicrobial resistant mutants compared to their respective wild-type. Values below 1 (horizontal dashed line) denote a relative decrease of the growth rate in the resistance mutant. For each strain, color represents resistance group (ciprofloxacin-resistant – pink, mecillinam-resistant – green, nitrofurantoin-resistant – gold, trimethoprim-resistant – purple) and symbols visualize the resistant mutants with efflux-related mutations (AcrAB-TolC + MdtK – circle, MdtK – diamond, EmrAB-TolC – square, or no efflux mutations – triangle). Error bars indicate 95% confidence interval of at least 8 replicates (≥ 3 biological replicates x 3 technical replicates were tested). In general, resistance to ciprofloxacin or mecillinam severely reduces the growth rate, whereas mutants resistant to nitrofurantoin and trimethoprim display low- to no apparent cost of resistance. Efflux mutations were mainly observed in isolates resistant to ciprofloxacin and nitrofurantoin.

Supplementary Figure 4

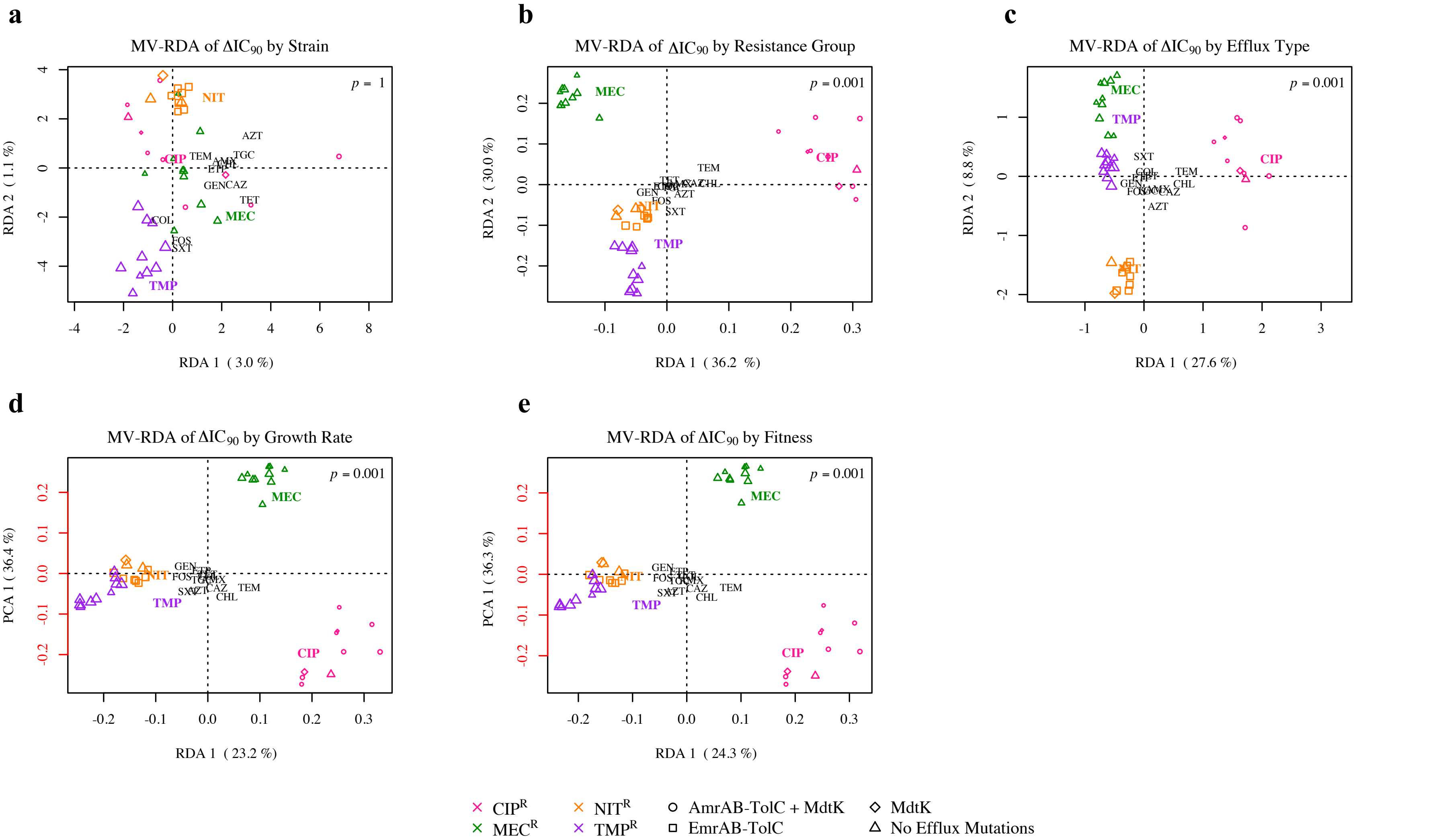

Supplementary Figure 5

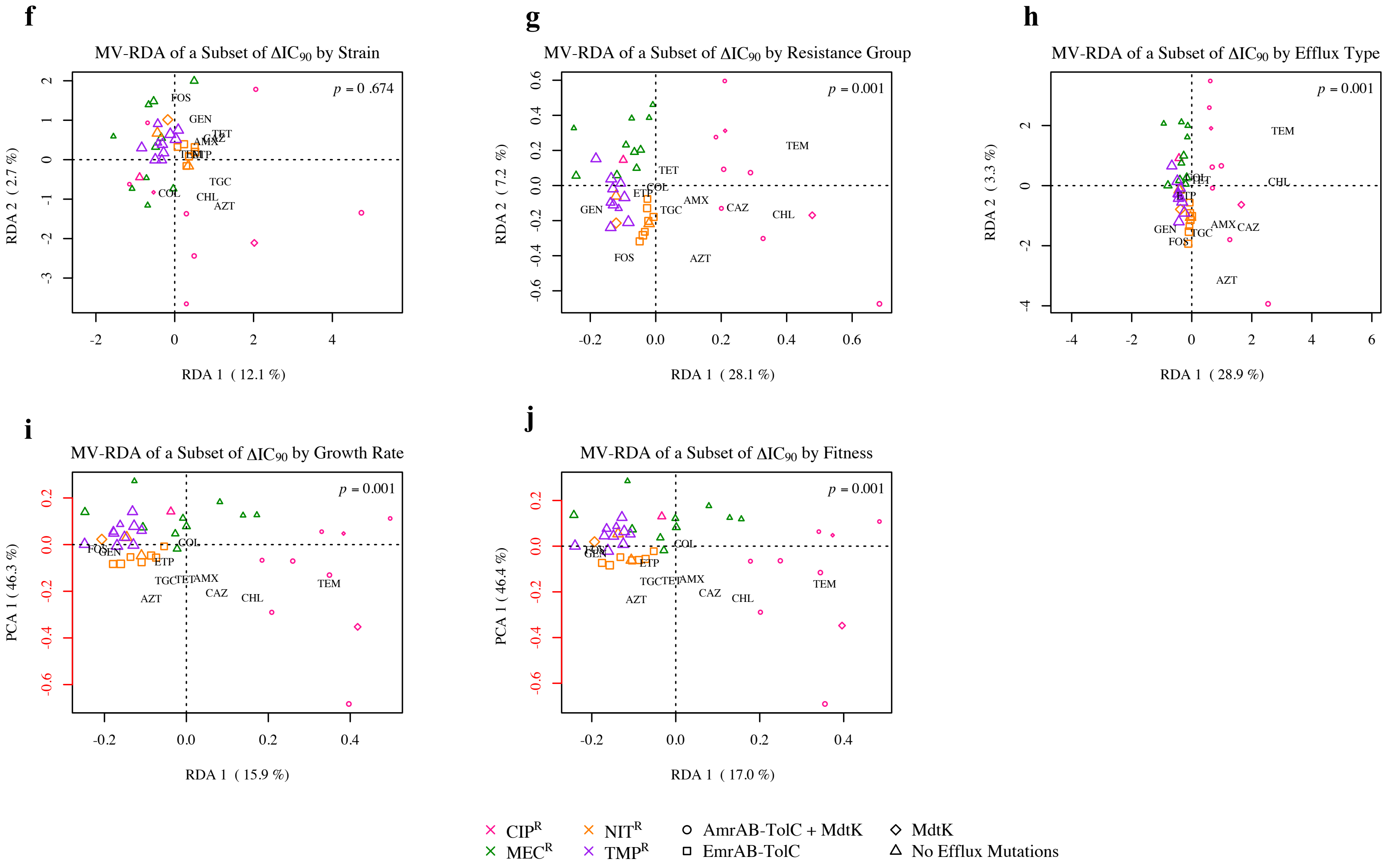

Supplementary Figure 5

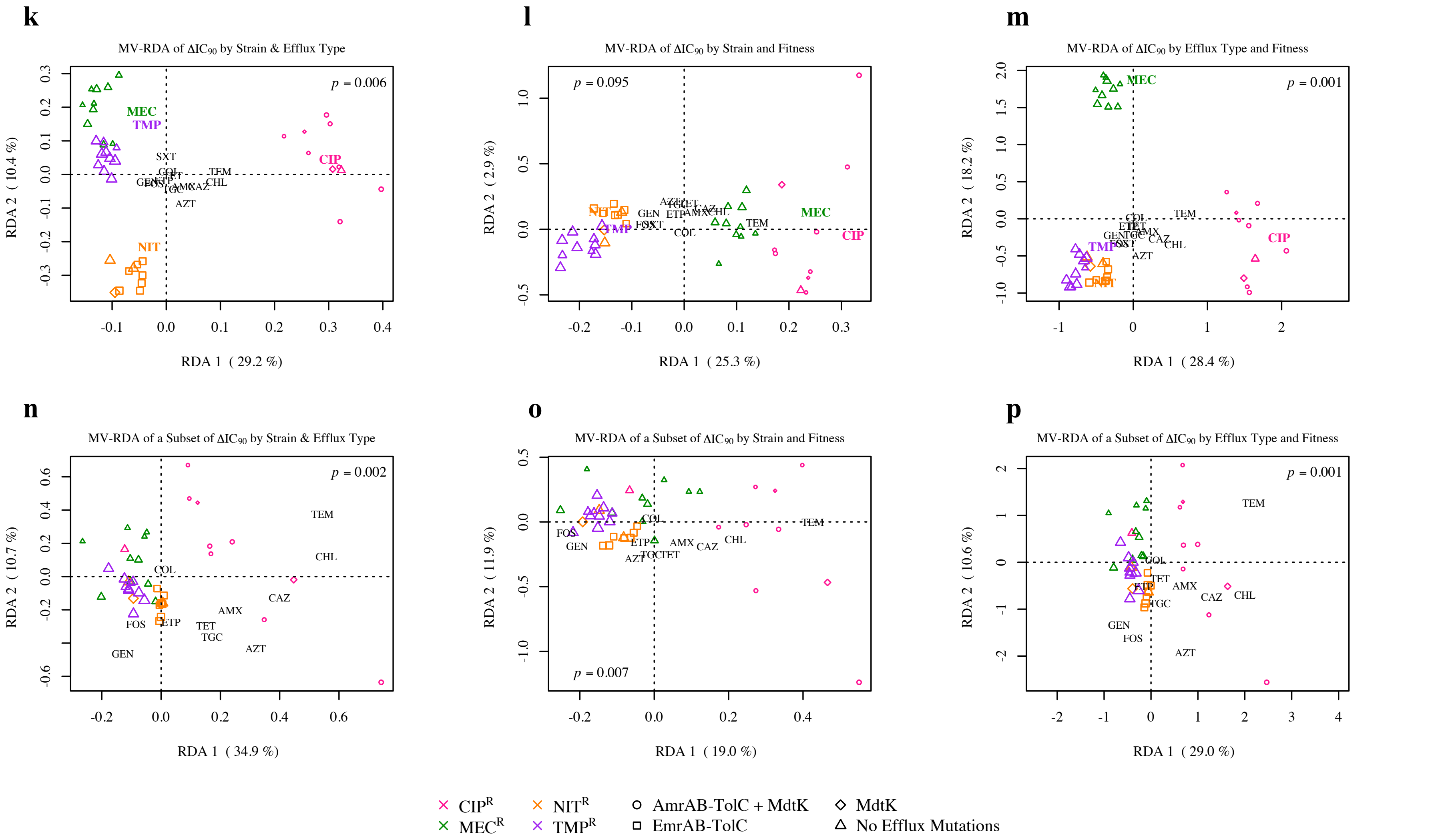

Supplementary Figure 5

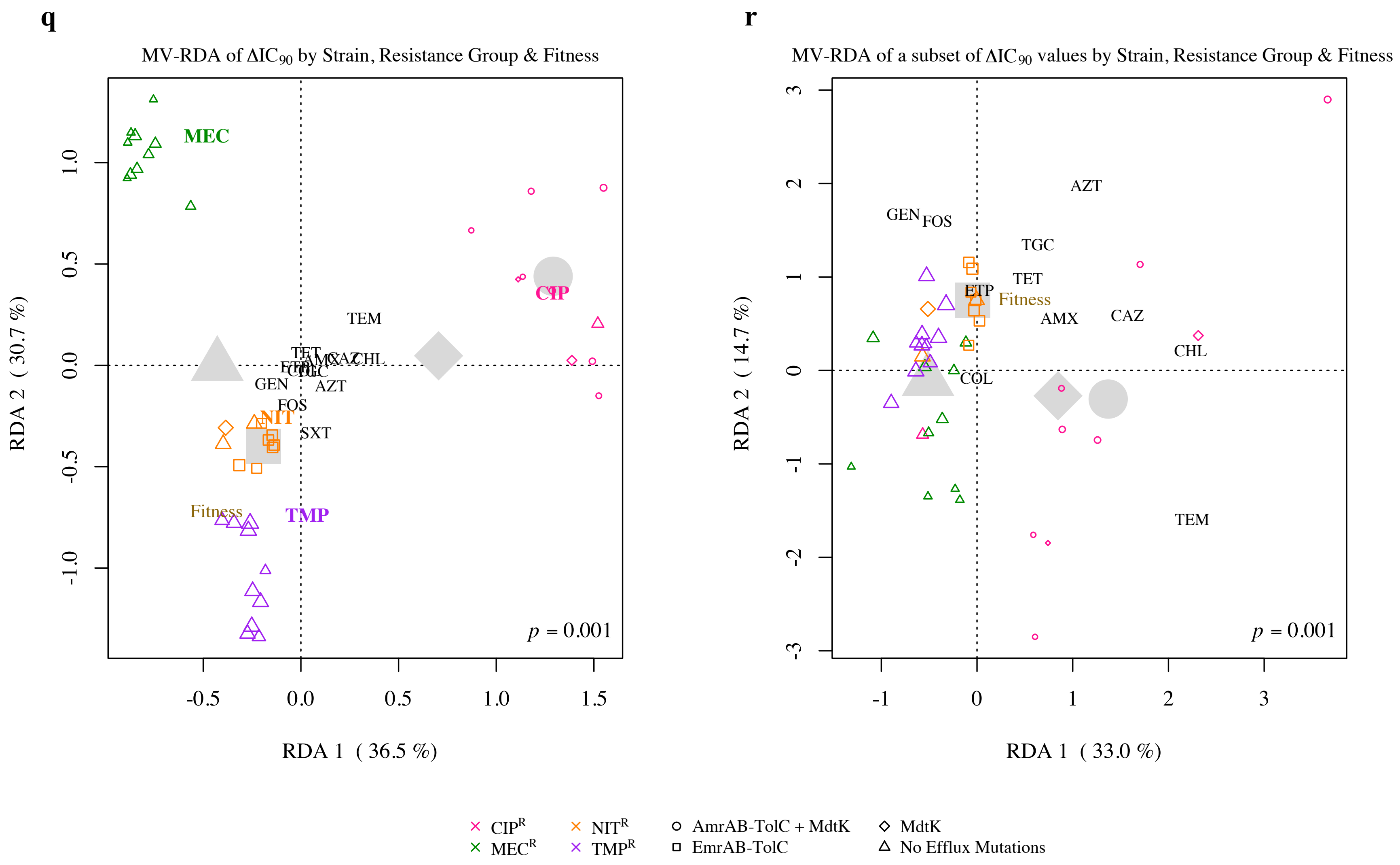

Supplementary Figure 5

Supplementary Figure 5. **Multivariate models of individual and combinations of factors.** (**a-e**) Graphical representations showing results of redundancy analyses (RDA, triplot) relating individual factors (strain background, resistance group, presence of efflux-related mutations [efflux type], growth rate and relative fitness) to the observed changes in IC_{90} between antimicrobial resistant mutants and the respective wild-type strain for 16 antimicrobials. And **f-j** displays the same analyses for a subset of the antimicrobials excluding ciprofloxacin, mecillinam, nitrofurantoin, trimethoprim and trimethoprim-sulfamethoxazole. Two-factor RDAs for both the total (**k-m**) and subset (**n-p**) of IC_{90} data were performed using combinations of factors (strain background, efflux type, and relative fitness). Resistance group and growth rate factors were excluded from combinatory analyses due to the co-linearity of growth rate and relative fitness and the greater biological relevance of resistance mechanisms (efflux types) than resistance group. The final three-factor models (Fig. 3) and three factors models using resistance group instead of efflux type for total (**q**) and subset (**r**) of IC_{90} data, were highly similar. RDA significance was assessed by permutation test (1000 permutations) where $p \leq 0.05$ was considered significant. For interpretation, the weighted average of each antimicrobial resistance mutant is plotted as a single colored symbol along the first and second axes, where color indicates the resistance group (ciprofloxacin-resistant – pink, mecillinam-resistant – green, nitrofurantoin-resistant – gold, trimethoprim-resistant – purple) and symbol size is proportional to the relative fitness, where smaller size indicates a greater reduction in growth rate compared to the wild-type. For panels **a-r**, shape represents the assigned efflux mutation group (circle – AcrAB-TolC + MdtK, square – EmrAB-TolC, diamond – MdtK, triangle – no efflux-related mutations). For panels **q-r**, Large grey symbols show the centroids (average effect) for all resistant mutants within a given efflux group (shape). Antimicrobial drug names indicate the tip of vectors that pass through the origin in the direction of increasing IC_{90} fold change or CR (direction of steepest ascent). These vectors can be used to interpret the change in IC_{90} for any of the antimicrobials shown. Redundancy analyses performed with single factor continuous data (panels **a-j**, on growth rates and relative fitness) resulted in only one constrained (RDA) axis (black), the second axis is instead the unconstrained (principle component analysis, PCA) axis (red), in these cases only the distribution along the first axis is relevant.

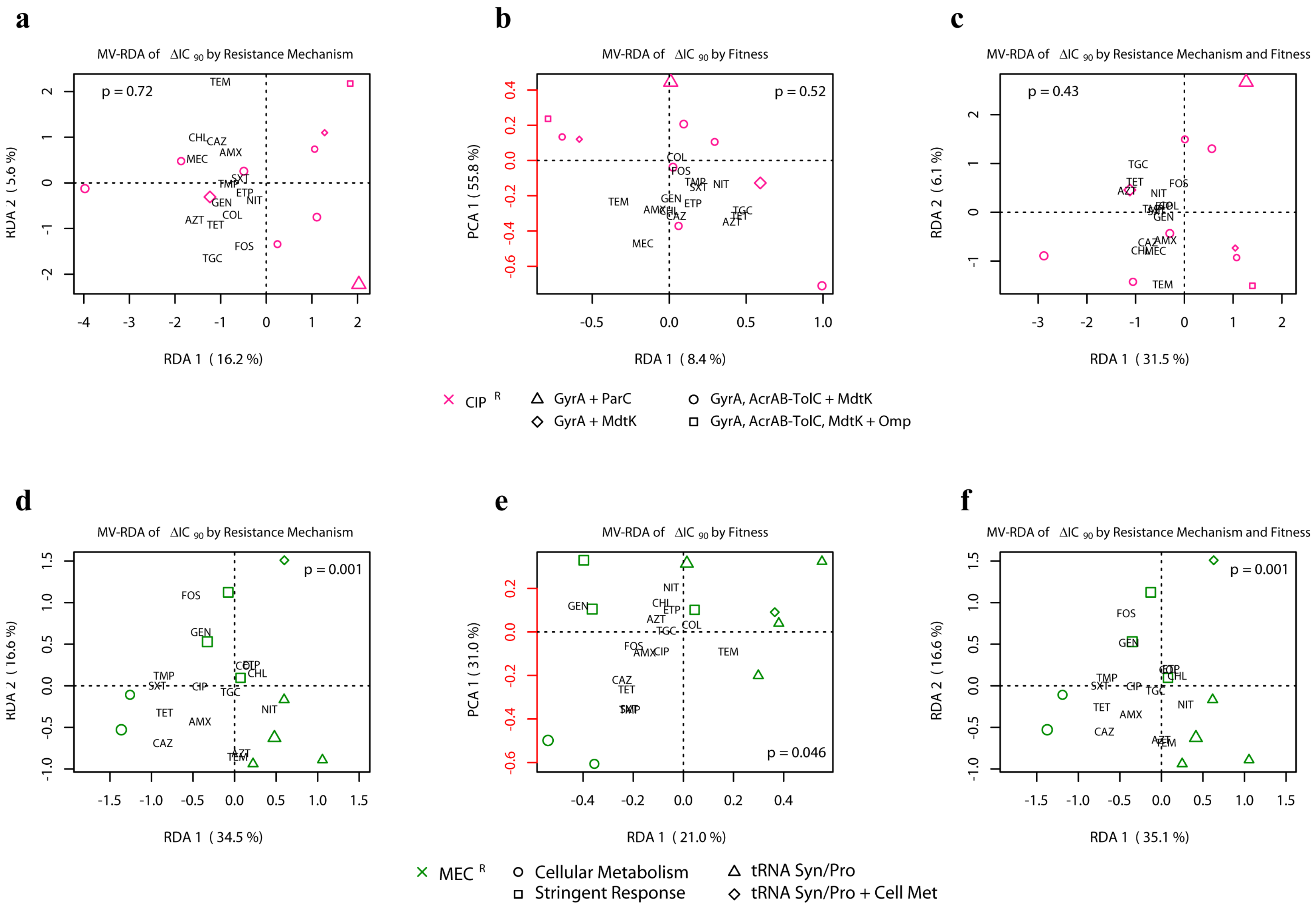

Supplementary Figure 6

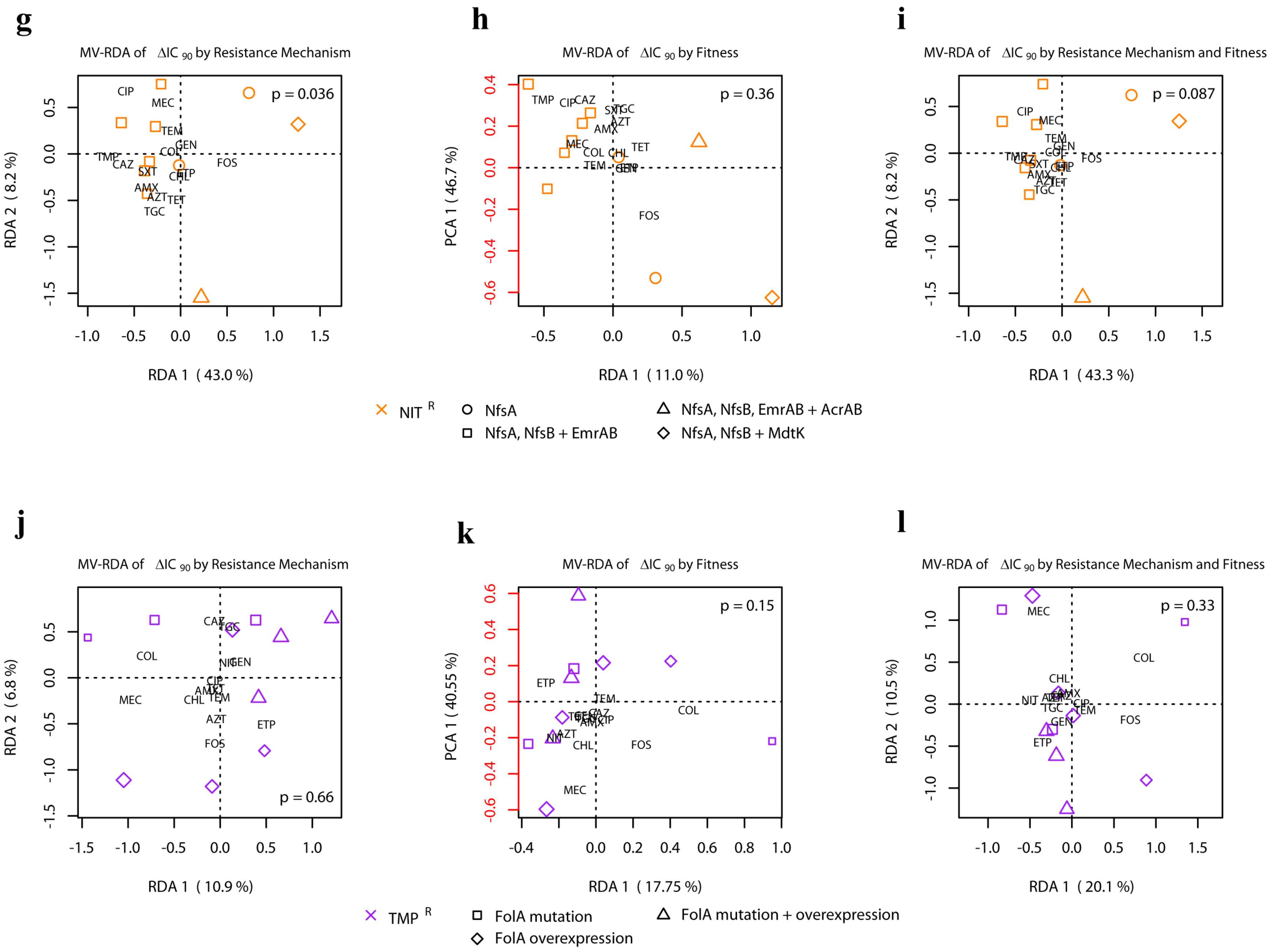

Supplementary Figure 6

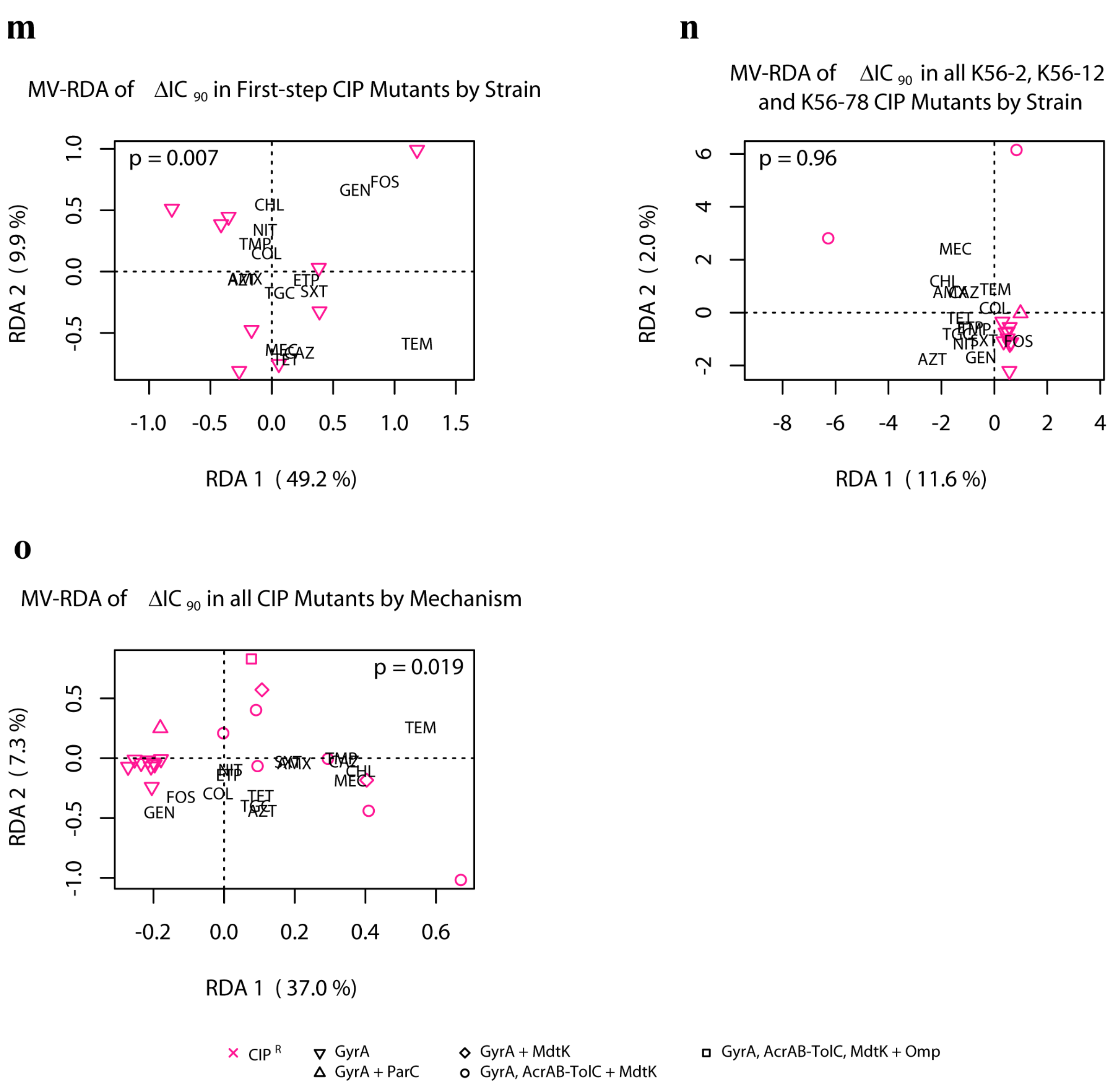

Supplementary Figure 6

Supplementary Figure 6. Multivariate models of the influence of resistance mechanisms on IC_{90} variation. For each resistance group, redundancy analyses were performed with antimicrobial resistance mechanism and relative fitness as factors (**a-l**). Redundancy analyses were also performed on nine first-step ciprofloxacin mutants with and without other ciprofloxacin-resistant mutants from the same strain backgrounds, K56-2, K56-12 and K56-78 (**m-o**). Resistance mechanism could not be assessed in these smaller sub-groupings due to the low number of mutants representing each mechanism, but an analysis was performed on all ciprofloxacin-resistant and the first-step ciprofloxacin mutants (**o**) which showed a significant contribution of resistance mechanism, contrary to the result when looking at the ciprofloxacin-resistant mutants alone (**a-c**). RDA significance was assessed as for Supp. Fig. 5. The weighted average of each antimicrobial resistance mutant is plotted as a single colored symbol along the first and second axes, where color indicates the resistance group (ciprofloxacin-resistant – pink, mecillinam-resistant – green, nitrofurantoin-resistant – gold, trimethoprim-resistant – purple) and symbol size is proportional to the relative fitness, where smaller size indicates a greater reduction in growth rate compared to the wild-type. For panels **a-o-**shape is unique for each resistance group and represents a detailed and drug-specific resistance mechanism (see individual panel legends).

Supplementary Table 1. **Gradient strip diffusion assay results.** Laboratory-selected antimicrobial resistant mutants were confirmed to be clinically-resistant to the respective antimicrobial used for selection at or above clinical breakpoint values when susceptibility was assessed by gradient strip diffusion testing, results represent a single measurement.

	Minimal inhibitory concentration ($\mu g\ mL^{-1}$)[1]							
	Ciprofloxacin		Mecillinam		Nitrofurantoin		Trimethoprim	
Strain	WT	CIP^R	WT	MEC^R	WT	NIT^R	WT	TMP^R
K56-2	0.023	> 32	0.25	> 256	16	256	0.38	> 32
K56-12	0.032	6	0.38	24	16	256	0.75	> 32
K56-16[2]	0.012	8	0.19	> 256	16	> 512	0.38	> 32
K56-41	0.023	4	0.19	16	16	> 512	0.25	> 32
K56-44[2]	0.023	3	0.19	32	12	256	0.38	16
K56-50	0.012	12	0.38	192	32	> 512	0.094	> 32
K56-68	0.023	> 32	0.25	64	16	> 512	0.5	> 32
K56-70	0.008	4	0.75	> 256	8	256	0.19	> 32
K56-75[3]	0.008	4	0.25	> 256	32	> 512	0.19	6
K56-78	0.016	16	0.38	16	32	256	0.38	24
Resistance Breakpoint[4]	> 0.5		> 8		> 64		> 4	

[1] Detection limit: ciprofloxacin = 32 $\mu g\ mL^{-1}$, mecillinam = 256 $\mu g\ mL^{-1}$, nitrofurantoin = 512 $\mu g\ mL^{-1}$, and trimethoprim = $\mu g\ mL^{-1}$.
[2, 3] Strains found to contain the Col156 or Col(MP18) replicon, respectively.
[4] EUCAST clinical breakpoint values for Enterobacteriaceae[1].

Supplementary Table 2. Tabulation of collateral responses detected in 40 antimicrobial resistant mutants, excluding primary resistance to drugs used for selection and trimethoprim-sulfamethoxazole for isolates resistant to trimethoprim.

	CIP^R	MEC^R	NIT^R	TMP^R	Total
Collateral sensitivity	38	41	3	10	92
Collateral sensitivity, ≥ 2 fold ΔIC_{90}	21	14	0	0	35
CS_{50}, collateral sensitivity ≥ 50% of strains	4	2	0	0	
Cross-resistance	70	21	37	13	141
Cross-resistance, ≥ 2 fold ΔIC_{90}	56	13	9	5	83
CR_{50}, collateral resistance ≥ 50% of strains	8	2	3	0	
Total collateral responses	108	62	40	23	233
Collateral responses, ≥ 2 fold ΔIC_{90}	77	27	9	5	118
CE_{50}, Collateral responses ≥ 50% of strains	12	4	3	0	
No change	42	88	110	117	357
Collateral responses tested	150	150	150	140	590
Total IC_{90}s tested	160	160	160	160	640

Abbreviations: ΔIC_{90} – change in inhibitory concentration 90% value (mutant vs. wild type).

Supplementary Table 3. **Summary of the output from multivariate models and permutation tests.** The explanatory value of different multivariate models (rows) and the individual factors included, strain background, presence of efflux-related mutations (efflux group) and relative fitness, on complete and a subset of antimicrobial susceptibility changes as determined by permutation tests.

Complete IC_{90} fold changes					Subset[1] of IC_{90} fold changes				
Factors[2]			Proportion of total variance explained[3]	Model	Factors[2]			Proportion of total variance explained[4]	Model
S	E	F	Inertia (%)	Sig.	S	E	F	Inertia (%)	Sig.
ns (1)	-	-	1.22569 (6.5%)	ns (1)	ns (0.667)	-	-	0.8236 (20.8%)	ns (0.674)
-	*** (0.001)	-	6.8920 (36.8%)	*** (0.001)	-	*** (0.001)	-	1.3098 (33.2%)	*** (0.001)
-	-	*** (0.001)	4.5463 (24.3%)	*** (0.001)	-	-	*** (0.001)	0.6730 (17.0%)	*** (0.001)
ns (1)	*** (0.001)	-	8.5189 (45.4%)	** (0.006)	ns (0.093)	*** (0.001)	-	2.1832 (55.3%)	** (0.002)
ns (1)	-	*** (0.001)	5.9273 (31.6%)	ns (0.095)	ns (0.317)	-	*** (0.001)	1.5197 (38.5%)	** (0.007)
-	*** (0.001)	*** (0.001)	9.8295 (52.4%)	*** (0.001)	-	*** (0.001)	** (0.006)	1.6224 (41.1%)	*** (0.001)
ns (0.993)	*** (0.001)	*** (0.001)	11.7274 (62.6%)	*** (0.001)	* (0.040)	*** (0.001)	** (0.009)	2.4180 (61.2%)	*** (0.001)

[1] A subset of IC_{90} fold changes, where IC_{90} data for ciprofloxacin, mecillinam, nitrofurantoin, trimethoprim and trimethoprim-sulfamethoxazole were excluded from all antimicrobial resistance groups.
[2] Factors used for and their significance by permutation test (1000 permutations) within a multivariate model. S – strain background, E – efflux group, F – fitness (relative growth rate), ns – not significant ($p > 0.05$), * $p \leq 0.05$, ** $p \leq 0.01$, *** $p \leq 0.001$. Factors excluded from a model are indicated with -.
[3,4] Total variance (inertia) in the IC_{90} fold change data for the complete model and the subset was 17.52443 and 3.1279, respectively.

Supplementary Table 4. Genomic characterization of *Escherichia coli* strains used in this study.

Strain	Origin[1]	Reported MLST[1]	BioSample[2]	Accession Number	MLST V1.8	PlasmidFinder V 1.3	ResFinder V 3.0
K56-2	Greece	ST-73	SAMN08095529 : K56-2	PHQR00000000	ST-73	no hits	no hits
K56-12	Portugal	ST-104	SAMN08095531 : K56-12	PIJI00000000	ST-104	no hits	no hits
K56-16	Portugal	ST-127	SAMN08095533 : K56-16	PIJJ00000000	ST-127	Col156	no hits
K56-41	Greece	ST-420	SAMN08095534 : K56-41	PIJK00000000	ST-73	no hits	no hits
K56-44	Greece	ST-12	SAMN08095535 : K56-44	PIJL00000000	ST-12	Col156	*sul2* [3]
K56-50	Greece	ST-100	SAMN08095551 : K56-50	PIJM00000000	ST-100	no hits	PmrB V161G[4]
K56-68	Sweden	ST-95	SAMN08095552 : K56-68	PIJN00000000	ST-95	no hits	no hits
K56-70	Sweden	ST-550	SAMN08095553 : K56-70	PIJO00000000	ST-537	no hits	PmrB V161G[4]
K56-75	UK	ST-69	SAMN08095554 : K56-75	PIJP00000000	ST-69	Col(MP18)	no hits
K56-78	UK	ST-1235	SAMN08095557 : K56-78	PIJQ00000000	ST-1235	no hits	ParE D475E[5]

[1] Reported information on *Escherichia coli* strains used in this study[2].
[2] BioSamples listed are all part of the NCBI BioProject: PRJNA419689.
[3] Gene linked to sulphonamide resistance.
[4,5] Mutations linked to colistin and quinolone resistance, respectively.

Supplementary References:

1. The European Committee on Antimicrobial Susceptibility Testing. Breakpoint tables for interpretation of MICs and zone diameters. Version 7.1, Available at http://www.eucast.org. (2017).

2. Bengtsson, S., Naseer, U., Sundsfjord, A., Kahlmeter, G., & Sundqvist, M. Sequence types and plasmid carriage of uropathogenic *Escherichia coli* devoid of phenotypically detectable resistance. *J Antimicrob Chemother* **67**, 69-73 (2012).
