# Supplementary material for: Conserved collateral antibiotic susceptibility networks in diverse clinical strains of Escherichia coli
Source: Nat Commun. 2018 Sep 10;9:3673. doi: 10.1038/s41467-018-06143-y (PMC6131505; doi:10.1038/s41467-018-06143-y)
Supplement: Supplementary file 3 — Description of Additional Supplementary Information [file 41467_2018_6143_MOESM3_ESM.pdf]

Description of Additional Supplementary Files

File Name: Supplementary Data 1
Description: Supplementary Data 1 contains DNA mutations identified by comparative whole genome sequencing of the ciprofloxacin-resistant mutants in this study: This EXCEL sheet contains a description of the mutations Columns C-H, and the assigned efflux group (Column K) and resistance mechanism (Column L) used in the statistical modelling.

File Name: Supplementary Data 2
Description: Supplementary Data 2 contains DNA mutations identified by comparative whole genome sequencing of the mecillinam-resistant mutants in this study: This EXCEL sheet contains a description of the mutations Columns C-H, and the assigned efflux group (Column K) and resistance mechanism (Column L) used in the statistical modelling.

File Name: Supplementary Data 3
Description: Supplementary Data 3 contains DNA mutations identified by comparative whole genome sequencing of the nitrofurantoin-resistant mutants in this study: This EXCEL sheet contains a description of the mutations Columns C-H, and the assigned efflux group (Column K) and resistance mechanism (Column L) used in the statistical modelling.

File Name: Supplementary Data 4
Description: Supplementary Data 4 contains DNA mutations identified by comparative whole genome sequencing of the trimethoprim-resistant mutants in this study: This EXCEL sheet contains a description of the mutations Columns C-H, and the assigned efflux group (Column K) and resistance mechanism (Column L) used in the statistical modelling.
